# Impact of silencing automated penicillin cross-reactivity alerts on perioperative antibiotic prescribing and surgical site infection rates

**DOI:** 10.1017/ice.2025.10311

**Published:** 2025-12

**Authors:** Michael J. Durkin, Joshua Nordman, Alice Bewley, Andrew Atkinson, Jonas Marschall, Helen Newland, Kimberly G. Blumenthal

**Affiliations:** 1 Division of Infectious Diseases, Washington University School of Medicine, St. Louis, MO, USA; 2 University of Arizona College of Medicine – Phoenix, Phoenix, AZ, USA; 3 BJC Healthcare, St. Louis, MO, USA; 4 Division of Rheumatology Allergy and Immunology, Massachusetts General Hospital, Harvard Medical School, Boston, MA, USA

## Abstract

We evaluated the impact of silencing penicillin cross-reactivity alerts on perioperative antibiotic prescribing and surgical site infections (SSIs) in 6 hospitals using an interrupted time series analysis. Silencing the alerts minimally increased cefazolin prescribing among penicillin allergy labeled patients (sensitivity analysis only; *P* = 0.03) and had no influence on SSIs (*P* = 0.32).

## Introduction

An estimated 32 million people have a reported penicillin allergy in the US; 95% of these can tolerate penicillins and other beta-lactams. Patients with listed penicillin allergies are more likely to receive alternative antibiotics which are associated with increased hospital costs, drug toxicities, and adverse outcomes^1^—including a 50% increase in surgical site infections (SSIs) and increased rates of *Clostridioides difficile* (C. diff), MRSA, and VRE.^2,3^

When a patient has a penicillin allergy listed on their profile, most electronic medical records (EMRs) will alert surgeons who try to prescribe cefazolin that a penicillin allergy exists, and that a cephalosporin is contraindicated. This changes prescribing practices: one study found 92% of patients without penicillin allergy received cefazolin compared to 12% of those with a charted penicillin allergy.^2^ Silencing of these alerts has led to increased cephalosporin prescribing without increasing adverse reactions.^4^ The purpose of this manuscript was to evaluate the impact of silencing a penicillin allergy cross-reactivity alert on the choice of perioperative antibiotic prophylaxis and SSI.

## Methods

A detailed description of silencing the penicillin cross-reactivity alert is in the supplement. For descriptive summaries, categorical variables were expressed as number (percentage) and median (interquartile range) for continuous variables. Differences in aggregated prescription proportions and surgical site infection rates before and after the intervention were investigated using a chi-square test. As primary analysis, we performed Poisson mixed effects interrupted time series analyses to evaluate the association between silencing penicillin allergy cross-reactivity alerts and a) antibiotic prescribing and b) SSI rates with BJC Healthcare, a 14 acute care hospital network in the St. Louis MO region. Models included the number of procedures as the denominator (offset) and accounted for intra-hospital correlation with a random effect for each hospital. The penicillin-cephalosporin allergy cross-reactivity alert was silenced in March 2021 for patients with non-serious penicillin labels within BJC Healthcare (“time 0”). We collected data for the 12 months prior to the intervention and 12 months after silencing the alert from patients receiving coronary artery bypass graft, colorectal, hip prosthesis, or hysterectomy procedures between March 1, 2020 and February 28, 2022 from six BJC hospitals that performed at least 10 procedures a month during this 24-month period. We considered only patients whose antibiotic were administered prophylactically and therefore started prior to incision. All procedures and surgical site infections were prospectively captured using National Healthcare Safety Network Surveillance definitions.^5^ Patients without a listed penicillin allergy served as controls to evaluate for general temporal trends.

Cefazolin prescribing and SSI rates were compared separately before and after the alert was silenced by fitting Poisson models tracking the trajectory of the endpoints over time separately for penicillin and non-penicillin allergy labeled patients. A sensitivity analysis for cefazolin prescribing with a 3-month interruption lead was also performed due to education occurring in the months before the alert was silenced. Details of the education are described in the supplement. Models were checked for autocorrelation via the autocorrelation function and no substantial autocorrelation was detected. We also evaluated cefazolin prescribing and surgical site infection rates stratified by hospital and surgery type to identify heterogeneity in the impact of the intervention.

## Results

6,204 patients were included. Most patients were female (62%), white (83.4%), and did not have a charted penicillin allergy (85%). Hip prosthesis and colorectal surgeries were most common (39% and 31%, respectively), followed by hysterectomies (16%) and coronary artery bypass grafts (14%). Among the 215 surgical site infections, 96 (45%) were intraabdominal. Cefazolin prescribing increased from 33% to 39% among penicillin allergy labeled patients but was unchanged in the control group at 55% for both time periods (supplement table 1).

**Figure 1.**
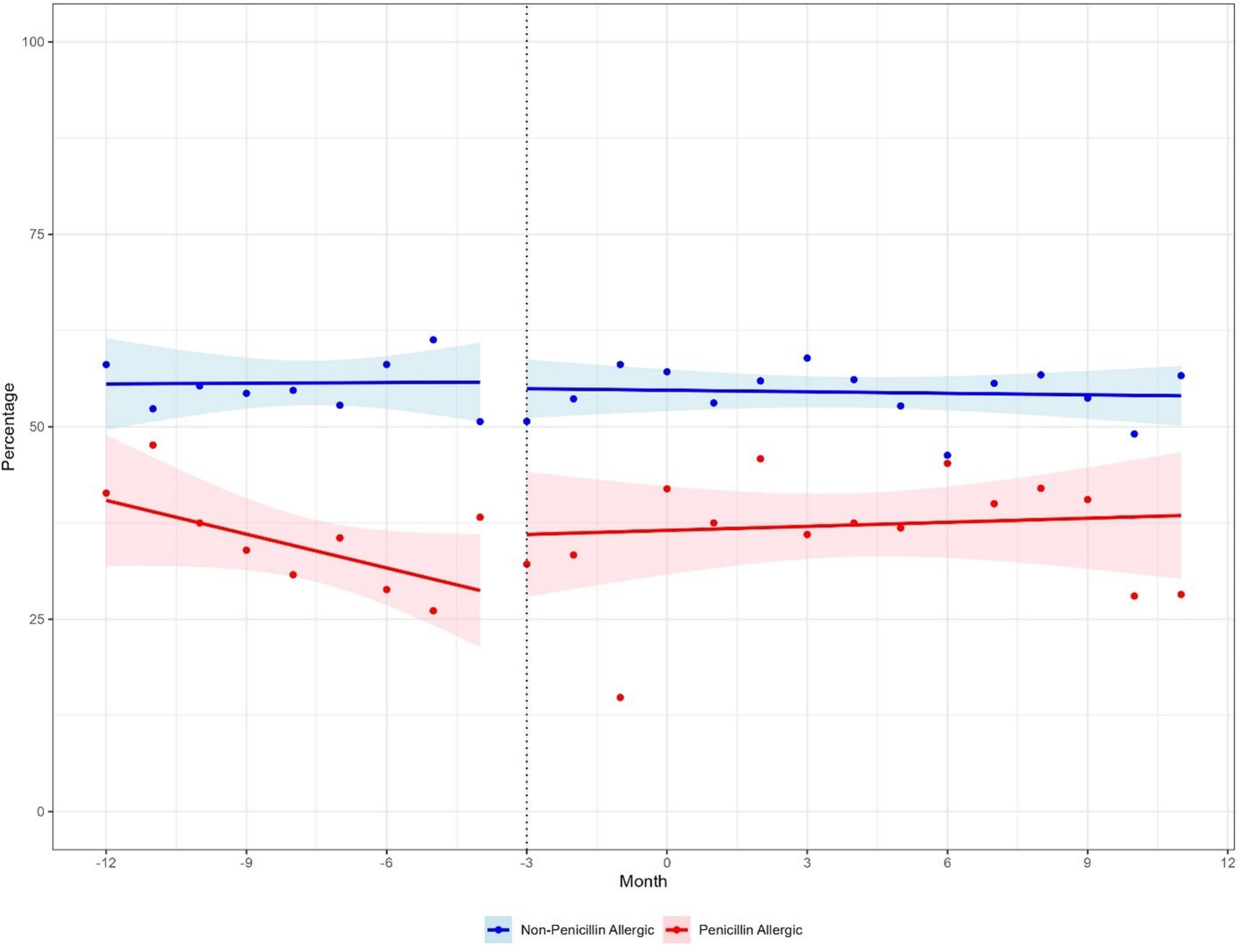
Sensitivity analysis for cefazolin prescribing using alternative start time on penicillin allergy cross reactivity alerts.

The slopes of cefazolin prescribing in both penicillin and non-penicillin allergy labeled patients did not significantly change in the post-intervention period when using March 2021 as the intervention in the interrupted time series analysis (*P*-value for change in slope pre/post = 0.38 (penicillin allergy labeled patients), *P* = 0.56 (controls)). However, when considering the sensitivity analysis including a 3-month lead time for the start of the intervention, the rate of cefazolin prescribing significantly increased in reportedly penicillin allergy labeled patients (*P* = 0.03), but not in the control patients (*P* = 0.71) (Fig. 1). Although SSI rates did not significantly change in either group after the alert was silenced when compared to the 12 months prior (penicillin allergy labeled 2.9% before versus 4.0% after, *P* = 0.32, controls 3.1% versus 3.3%, *P* = 0.99), there was a noticeable change in the trend of the SSI rate (Figure S1).

Stratified results are displayed in the supplement. Specifically, results showing antibiotic prescribing rates by surgery type and hospital are displayed in supplemental tables 2 and 3, respectively. These showed larger increases in cefazolin prescribing for coronary artery bypass grafts and hysterectomies and smaller increases for colorectal surgery and knee replacements. Stratified results by hospital also showed substantial heterogeneity in cefazolin prescribing before vs after the intervention. Surgical site infection rates stratified by procedure and hospital are also displayed in supplemental tables 4 and 5, respectively.

## Discussion

This study identified that silencing penicillin allergy cross-reactivity alerts rates modestly increased cefazolin prescribing. To our knowledge, this is the second study to evaluate the impact of silencing penicillin allergy cross-reactivity alerts on perioperative antibiotic prescribing and the first to evaluate its impact on surgical site infection rates.

Overall, we only observed a 6% increase in cefazolin prescribing after silencing the cross-reactivity alerts—despite broad educational interventions that accompanied the silencing of the alert. These results are much more modest than those found by Bogus et al, where they observed an increase from 50% to 74%.^6^ Our data demonstrated that there is substantial variability in uptake between institutions and surgical specialty. We also noted more substantial increases in cefazolin prescribing among select hospitals within our network. Hospitals that are exploring silencing penicillin allergy cross-reactivity alerts should consider additional implementation science strategies to increase cefazolin prescribing. Additional studies should explore why these barriers exist and how to overcome them, as previous data has shown that silencing these alerts are safe.^7,8^

There are limitations to this study. The number of National Healthcare Safety Network (NHSN)-defined surgeries that were performed in both the pre- and post-period of the study restricted the sample size. A large number of colorectal surgeries were included in the dataset; SSIs from these surgeries often arise from anastomotic leaks, which may not be as preventable with SSI prophylaxis as those caused by skin bacteria. There are likely additional benefits to silencing penicillin allergy alerts such as improved operating room workflow (e.g., cefazolin is faster to infuse than vancomycin); however, these were not explored in our analyses.

## Conclusions

Our findings showed that a simple intervention, such as adjustments to EMR alerts, might lead changes toward safer perioperative antibiotic prescribing. However, additional strategies are likely needed to further optimize perioperative antibiotic prescribing. These strategies may need to be multifaceted, including collaborating with surgical colleagues and quality improvement groups to achieve larger changes in perioperative prescribing.

## Supporting information

Durkin et al. supplementary material Durkin et al. supplementary material

## Supplementary material

To view supplementary material for this article, please visit https://doi.org/10.1017/ice.2025.10311
