# Supplementary material for: Impact of silencing automated penicillin cross-reactivity alerts on perioperative antibiotic prescribing and surgical site infection rates
Source: Infect Control Hosp Epidemiol. 2025 Sep 29;46(12):1265–7. doi: 10.1017/ice.2025.10311 (PMC12779457; doi:10.1017/ice.2025.10311)

**Supplement:**

**Supplement Table 1.** Patient Characteristics

| PATIENTS | | | | | |
| --- | --- | --- | --- | --- | --- |
| Characteristic, n (%) | All (n = 6204) | Control, Pre-Intervention (n = 2632) | Control, Post-Intervention (n = 2648) | Penicillin Allergy Labelled, Pre-Intervention (n = 438) | Penicillin Allergy Labelled, Post-Intervention (n = 486) |
| Age, median (IQR) | 66 (55, 74) | 65 (55, 74) | 66 (56, 74) | 65 (55, 73) | 65 (55, 73) |
| Female sex | 3837 (61.9) | 1581 (60.1) | 1623 (61.3) | 296 (67.7) | 337 (69.3) |
| Race |  |  |  |  |  |
| White | 5172 (83.4) | 2215 (84.2) | 2187 (82.6) | 366 (83.6) | 404 (83.1) |
| Black | 948 (15.3) | 377 (14.3) | 430 (16.2) | 63 (14.4) | 78 (16.0) |
| Asian | 33 (0.5) | 18 (0.7) | 11 (0.4) | 4 (0.9) | 0 (0.0) |
| Other | 12 (0.2) | 5 (0.2) | 4 (0.2) | 2 (0.5) | 1 (0.2) |
| Unknown | 39 (0.6) | 17 (0.6) | 16 (0.6) | 3 (0.7) | 3 (0.6) |
| Ethnicity |  |  |  |  |  |
| Hispanic | 80 (1.3) | 30 (1.1) | 33 (1.2) | 6 (1.4) | 11 (2.3) |
| Non-Hispanic | 6076 (97.9) | 2577 (97.9) | 2597 (98.1) | 430 (98.2) | 472 (97.1) |
| Unknown | 48 (0.8) | 25 (0.9) | 18 (0.7) | 2 (0.5) | 3 (0.6) |
| Body Mass Index |  |  |  |  |  |
| <18.5 | 135 (2.2) | 46 (1.7) | 63 (2.4) | 11 (2.5) | 15 (3.1) |
| 18.5-24.9 | 1423 (22.9) | 605 (23.0) | 613 (23.1) | 91 (20.8) | 114 (23.5) |
| 25.0-29.9 | 1853 (29.9) | 800 (30.4) | 797 (30.1) | 132 (30.1) | 124 (25.5) |
| ≥30.0 | 2793 (45.0) | 1181 (44.9) | 1175 (44.4) | 204 (46.6) | 233 (47.9) |
| ASA Class |  |  |  |  |  |
| I | 49 (0.8) | 32 (1.2) | 14 (0.5) | 1 (0.2) | 2 (0.4) |
| II | 2104 (33.9) | 933 (35.4) | 846 (31.9) | 167 (38.1) | 158 (32.5) |
| III | 2943 (47.4) | 1213 (46.1) | 1288 (48.6) | 196 (44.7) | 246 (50.6) |
| IV | 1081 (17.4) | 444 (16.9) | 491 (18.5) | 73 (16.7) | 73 (15.0) |
| V | 27 (0.4) | 10 (0.4) | 9 (0.3) | 1 (0.2) | 7 (1.4) |
| Medical comorbidities |  |  |  |  |  |
| Diabetes | 1767 (28.5) | 720 (27.4) | 786 (29.7) | 129 (29.5) | 132 (27.2) |
| Cardiovascular disease | 1266 (20.4) | 512 (19.5) | 570 (21.5) | 95 (21.7) | 89 (18.3) |
| Malignancy | 719 (11.6) | 307 (11.7) | 301 (11.4) | 56 (12.8) | 55 (11.3) |
| Chronic pulmonary disease | 1823 (29.4) | 742 (28.2) | 790 (29.8) | 130 (29.7) | 161 (33.1) |
| Chronic renal impairment | 1326 (21.4) | 548 (20.8) | 583 (22.0) | 95 (21.7) | 100 (20.6) |
| Cardiac arrhythmias | 2545 (41.0) | 1086 (41.3) | 1072 (40.5) | 180 (41.1) | 207 (42.6) |
| Valvular disease | 1552 (25.0) | 634 (24.1) | 678 (25.6) | 112 (25.6) | 128 (26.3) |
| Pulmonary circulation disorders | 645 (10.4) | 283 (10.8) | 270 (10.2) | 43 (9.8) | 49 (10.1) |
| Peripheral vascular disorders | 1519 (24.5) | 641 (24.4) | 672 (25.4) | 105 (24.0) | 101 (20.8) |
| Paralysis | 163 (2.6) | 61 (2.3) | 76 (2.9) | 14 (3.2) | 12 (2.5) |
| Neurodegenerative disorders | 776 (12.5) | 322 (12.2) | 326 (12.3) | 63 (14.4) | 65 (13.4) |
| Hypothyroidism | 1709 (27.5) | 705 (26.8) | 737 (27.8) | 130 (29.7) | 137 (28.2) |
| Liver disease | 980 (15.8) | 382 (14.5) | 443 (16.7) | 71 (16.2) | 84 (17.3) |
| Peptic ulcer disease excluding bleeding | 221 (3.6) | 86 (3.3) | 92 (3.5) | 24 (5.5) | 19 (3.9) |
| AIDS/HIV | 25 (0.4) | 9 (0.3) | 12 (0.5) | 0 (0.0) | 4 (0.8) |
| Lymphoma | 95 (1.5) | 43 (1.6) | 42 (1.6) | 4 (0.9) | 6 (1.2) |
| Solid tumor without metastasis | 1770 (28.5) | 735 (27.9) | 754 (28.5) | 141 (32.2) | 140 (28.8) |
| Rheumatoid arthritis | 653 (10.5) | 259 (9.8) | 280 (10.6) | 36 (8.2) | 78 (16.0) |
| Coagulopathy | 984 (15.9) | 385 (14.6) | 447 (16.9) | 66 (15.1) | 86 (17.7) |
| Obesity | 2605 (42.0) | 1091 (41.5) | 1125 (42.5) | 183 (41.8) | 206 (42.4) |
| Weight loss | 993 (16.0) | 431 (16.4) | 427 (16.1) | 66 (15.1) | 69 (14.2) |
| Fluid and electrolyte disorders | 2633 (42.4) | 1082 (41.1) | 1158 (43.7) | 184 (42.0) | 209 (43.0) |
| Blood loss anemia | 396 (6.4) | 155 (5.9) | 187 (7.1) | 25 (5.7) | 29 (6.0) |
| Deficiency anemia | 856 (13.8) | 352 (13.4) | 370 (14.0) | 60 (13.7) | 74 (15.2) |
| Alcohol abuse | 269 (4.3) | 101 (3.8) | 128 (4.8) | 19 (4.3) | 21 (4.3) |
| Drug abuse | 277 (4.5) | 118 (4.5) | 118 (4.5) | 16 (3.7) | 25 (5.1) |
| Psychoses | 97 (1.6) | 40 (1.5) | 39 (1.5) | 6 (1.4) | 12 (2.5) |
| Depression | 1886 (30.4) | 749 (28.5) | 856 (32.3) | 137 (31.3) | 144 (29.6) |
| Hypertension | 4374 (70.5) | 1822 (69.2) | 1917 (72.4) | 309 (70.5) | 326 (67.1) |
| Drug allergies |  |  |  |  |  |
| Cephalosporin | 5 (0.1) | 1 (0.0) | 2 (0.1) | 0 (0.0) | 2 (0.4) |
| Vancomycin | 17 (0.3) | 8 (0.3) | 8 (0.3) | 0 (0.0) | 1 (0.2) |
| Total number of other allergies |  |  |  |  |  |
| 0 | 1240 (20.0) | 534 (20.3) | 490 (18.5) | 104 (23.7) | 112 (23.0) |
| 1-2 | 2315 (37.3) | 998 (37.9) | 1036 (39.1) | 140 (31.9) | 141 (29.0) |
| 3-4 | 1263 (20.4) | 537 (20.4) | 553 (20.9) | 75 (17.1) | 98 (20.2) |
| ≥5 | 1386 (22.3) | 563 (21.4) | 569 (21.5) | 119 (27.3) | 135 (27.9) |

| PROCEDURES | | | | |  |
| --- | --- | --- | --- | --- | --- |
| Characteristic | All (n = 6627) | Control, Pre-Intervention (n = 2774) | Control, Post-Intervention (n = 2871) | Penicillin Allergy Labelled, Pre-Intervention (n = 453) | Penicillin Allergy Labelled, Post-Intervention (n = 529) |
| Surgery type |  |  |  |  |  |
| CBGB | 930 (14.0) | 367 (13.2) | 430 (15.0) | 60 (13.2) | 73 (13.8) |
| COLO | 2039 (30.8) | 853 (30.7) | 903 (31.5) | 128 (28.3) | 155 (29.3) |
| HPRO | 2572 (38.8) | 1107 (39.9) | 1084 (37.8) | 172 (38.0) | 209 (39.5) |
| HYST | 1086 (16.4) | 447 (16.1) | 454 (15.8) | 93 (20.5) | 92 (17.4) |
| Emergency | 549 (8.3) | 240 (8.7) | 245 (8.5) | 26 (5.7) | 38 (7.2) |
| Trauma | 325 (4.9) | 157 (5.7) | 124 (4.3) | 23 (5.1) | 21 (4.0) |
| PATOS | 26 (0.4) | 10 (3.6) | 11 (3.8) | 2 (0.4) | 3 (0.6) |
| Wound class |  |  |  |  |  |
| Clean/Clean-contaminated | 5988 (90.4) | 2508 (90.4) | 2579 (89.8) | 412 (90.9) | 489 (92.4) |
| Contaminated/Dirty | 639 (9.6) | 266 (9.6) | 292 (10.2) | 41 (9.1) | 40 (7.6) |
| Procedure duration, median (IQR) | 125 (83, 214) | 115 (78, 201) | 134 (87, 225) | 119 (80, 215) | 128 (85, 215) |
| Received cefazolin | 3455 (52.1) | 1534 (55.3) | 1567 (54.6) | 148 (32.7) | 206 (38.9) |
| Received beta-lactam | 5451 (82.3) | 2304 (83.1) | 2408 (83.9) | 324 (71.5) | 415 (78.4) |
| Time between cefazolin and surgery, median (IQR) | -82 (-108, -51) | -84 (-108, -53) | -82 (-110, -50) | -79 (-96, -41) | -74 (-104, -39) |
| Time between first antibiotic and surgery, median (IQR) | -91 (-116, -66) | -89 (-112, -65) | -95 (-119, -67) | -87 (-106, -62) | -93 (-120, -70) |

| SURGICAL SITE INFECTIONS | | | | | |
| --- | --- | --- | --- | --- | --- |
| Characteristic | All (n = 215) | Control, Pre-Intervention (n = 85) | Control, Post-Intervention (n = 96) | Penicillin Allergy Labelled, Pre-Intervention (n = 13) | Penicillin Allergy Labelled, Post-Intervention (n = 21) |
| Infection Type |  |  |  |  |  |
| BONE – Osteomyelitis | 3 (1.4) | 1 (1.2) | 2 (2.1) | 0 (0.0) | 0 (0.0) |
| DIP – Deep Incisional Primary | **18 (8.4)** | **9 (10.6)** | **6 (6.2)** | **0 (0.0)** | **3 (14.3)** |
| DIS – Deep Incisional Secondary | 1 (0.5) | 1 (1.2) | 0 (0.0) | 0 (0.0) | 0 (0.0) |
| GIT – Gastrointestinal tract | 1 (0.5) | 0 (0.0) | 1 (1.0) | 0 (0.0) | 0 (0.0) |
| IAB – Intraabdominal, not specified elsewhere | **96 (44.7)** | **35 (41.2)** | **44 (45.8)** | **8 (61.5)** | **9 (42.9)** |
| MED – Mediastinitis | 2 (0.9) | 1 (1.2) | 0 (0.0) | 0 (0.0) | 1 (4.8) |
| OREP – Other infections of male/female reproductive tract | 2 (0.9) | 2 (2.4) | 0 (0.0) | 0 (0.0) | 0 (0.0) |
| PJI – Periprosthetic Joint Infection | 19 (8.8) | 12 (14.1) | 7 (7.3) | 0 (0.0) | 0 (0.0) |
| SIP – Superficial Incisional Primary | **70 (32.6)** | **24 (28.2)** | **34 (35.4)** | **5 (38.5)** | **7 (33.3)** |
| SIS – Superficial Incisional Secondary | 2 (0.9) | 0 (0.0) | 1 (1.0) | 0 (0.0) | 1 (4.8) |
| VCUF – Vaginal cuff | 1 (0.5) | 0 (0.0) | 1 (1.0) | 0 (0.0) | 0 (0.0) |

|  | Pre-Intervention | | Post-Intervention | |
| --- | --- | --- | --- | --- |
| Procedure Code | Penicillin Allergy | Control | Penicillin Allergy | Control |
| CBGB | 14 (23.3) | 269 (73.1) | 28 (38.4) | 338 (78.6) |
| COLO | 5 (3.9) | 39 (4.6) | 6 (3.9) | 52 (5.8) |
| HPRO | 85 (49.4) | 900 (81.3) | 114 (54.5) | 871 (80.4) |
| HYST | 44 (47.3) | 327 (73.2) | 58 (63.0) | 306 (67.4) |

**Supplement Table 2. Cefazolin receipt by procedure.**

N (%) of patients that received cefazolin prior to surgery incision time. CBGB = coronary artery bypass graft with both chest and donor site incisions, COLO = colon surgery, HPRO = hip prosthesis, HYST = abdominal hysterectomy.

**Supplement Table 3. Cefazolin receipt by hospital.**

|  | Pre-Intervention | | Post-Intervention | |
| --- | --- | --- | --- | --- |
| Hospital | Penicillin Allergy | Control | Penicillin Allergy | Control |
| AMH | 9 (36.0) | 85 (49.7) | 7 (41.2) | 76 (45.2) |
| BJH | 63 (35.6) | 587 (53.1) | 88 (39.3) | 574 (51.2) |
| BJSPH | 16 (42.1) | 106 (56.4) | 9 (33.3) | 71 (44.1) |
| BJWCH | 26 (44.8) | 280 (62.9) | 39 (40.2) | 302 (60.3) |
| CH | 3 (9.7) | 43 (21.0) | 7 (17.1) | 74 (29.2) |
| MBMC | 31 (25.0) | 434 (65.7) | 56 (45.5) | 470 (70.3) |

N (%) of patients that received cefazolin prior to surgery incision time. AMH = Alton Memorial Hospital, BJH = Barnes-Jewish Hospital, BJSPH = Barnes-Jewish St. Peters Hospital, BJWCH = Barnes-Jewish West County Hospital, CH = Christian Hospital, MBMC = Missouri Baptist Medical Center.

**Supplement Table 4. Surgical site infection rates by procedure.**

|  | Pre-Intervention | | Post-Intervention | |
| --- | --- | --- | --- | --- |
| Procedure Code | Penicillin Allergy | Control | Penicillin Allergy | Control |
| CBGB | 1.7% | 4.1% | 2.7% | 1.9% |
| COLO | 8.6% | 6.3% | 9.0% | 7.5% |
| HPRO | 0.0% | 1.2% | 1.4% | 1.0% |
| HYST | 1.1% | 0.9% | 2.2% | 2.0% |

Surgical site infection rates by procedure. CBGB = coronary artery bypass graft with both chest and donor site incisions, COLO = colon surgery, HPRO = hip prosthesis, HYST = abdominal hysterectomy.

**Supplement Table 5. Surgical site infection rates by hospital.**

|  | Pre-Intervention | | Post-Intervention | |
| --- | --- | --- | --- | --- |
| Hospital | Penicillin Allergy | Control | Penicillin Allergy | Control |
| AMH | 0.0% | 0.0% | 0.0% | 0.0% |
| BJH | 5.1% | 5.2% | 4.9% | 5.4% |
| BJSPH | 0.0% | 2.1% | 7.4% | 4.3% |
| BJWCH | 0.0% | 0.9% | 1.0% | 1.6% |
| CH | 3.2% | 2.4% | 4.9% | 1.6% |
| MBMC | 2.4% | 2.4% | 4.1% | 2.5% |

Surgical site infection rates by hospital. AMH = Alton Memorial Hospital, BJH = Barnes-Jewish Hospital, BJSPH = Barnes-Jewish St. Peters Hospital, BJWCH = Barnes-Jewish West County Hospital, CH = Christian Hospital, MBMC = Missouri Baptist Medical Center.

**Supplement Figure 1 Surgical Site Infection trajectory**  **Using Start Time on Penicillin Allergy Cross Reactivity Alerts.**


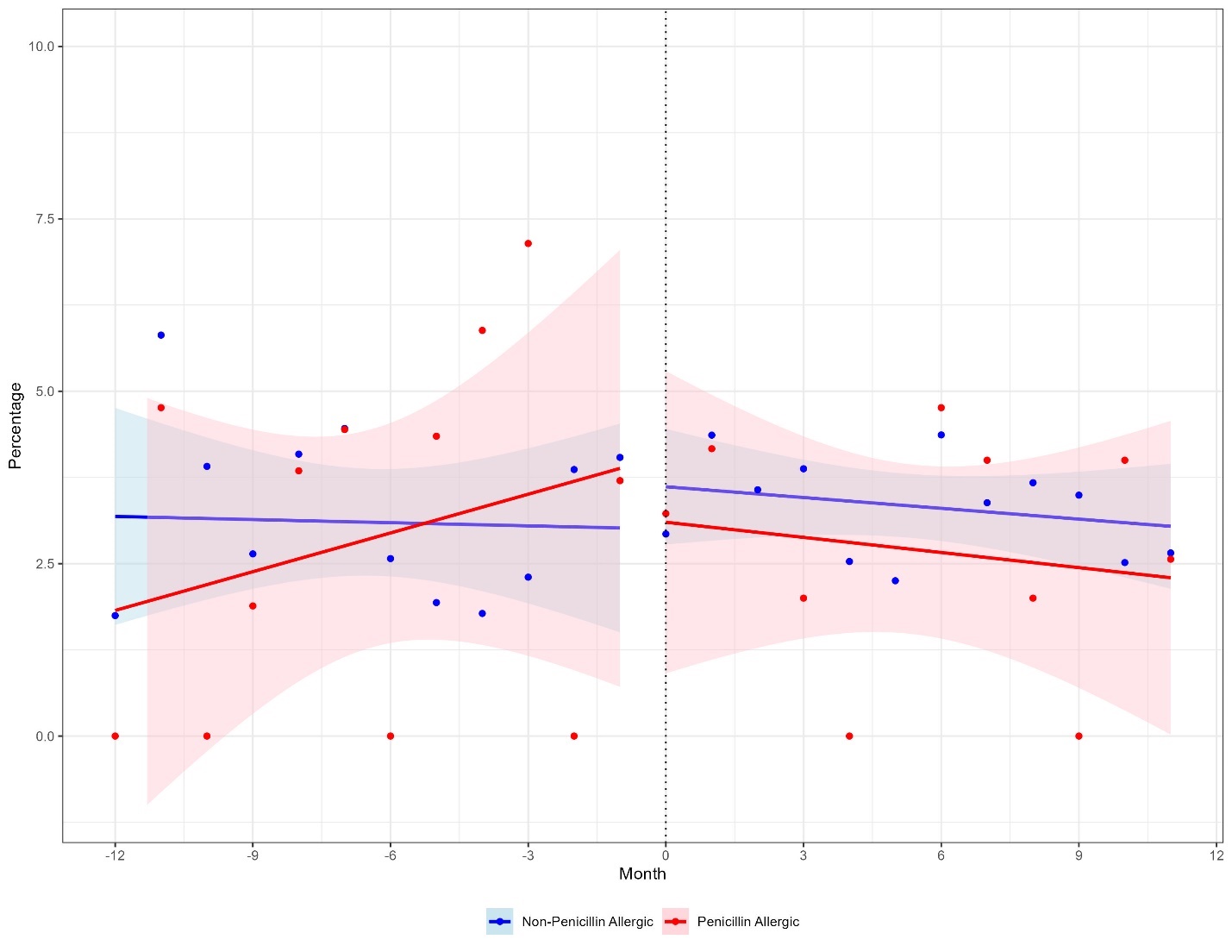


Interrupted time series analysis comparing surgical site infection percentages in penicillin allergy labelled patients (red dots) and non-penicillin allergy labelled patients (blue dots) before and after the alert was silenced. Month 0 indicates the first month the alert was silenced. An outlier hospital at month 2 for penicillin allergy labelled patients was excluded due to the low number of procedures in the respective month . Solid lines indicate linear models weighted by number of procedures performed, with 95% confidence intervals shaded.

**Description of silencing penicillin allergy cross reactivity alerts**

Penicillin allergy labels were classified as severe or not severe based on a multidisciplinary team of infectious disease doctors, pharmacists, and an allergist on the team. This was approved to the BJC Healthcare Pharmacy and Therapeutics Committee. We then required that healthcare providers document the nature of penicillin allergy labels to determine if a penicillin allergy could be silenced. Once an allergy label could be categorized, then we built a clinical decision support system to determine if the alert should be suppressed in Epic, through the Willow program.

Educational materials included email reminders, a teleconference, and educational handouts.

An example of which alerts were suppressed were provided to all healthcare providers via email, townhall meetings, and presentations to key stakeholder groups. Recipients include attending physicians, fellows, residents, pharmacists, and nurses. Supplement Figure 2 depicts a slide with the educational materials and which alerts were suppressed.

Please note that this intervention only applies to cross-reactivity alerts, so patients with a penicillin allergy label who were ordered an antibiotic in the penicillin family would still receive the original pop-up alert. Similarly, the penicillin cross reactivity alert applied to all penicillin-based antibiotics, rather than just penicillin alone (this included amoxicillin, ampicillin, dicloxacillin, nafcillin, oxacillin, penicillin, and piperacillin).

**Supplement Figure 2. An example of educational materials to healthcare providers outlining which penicillin allergy labels were suppressed based on the nature of the allergy and the approval process within BJC HealthCare**


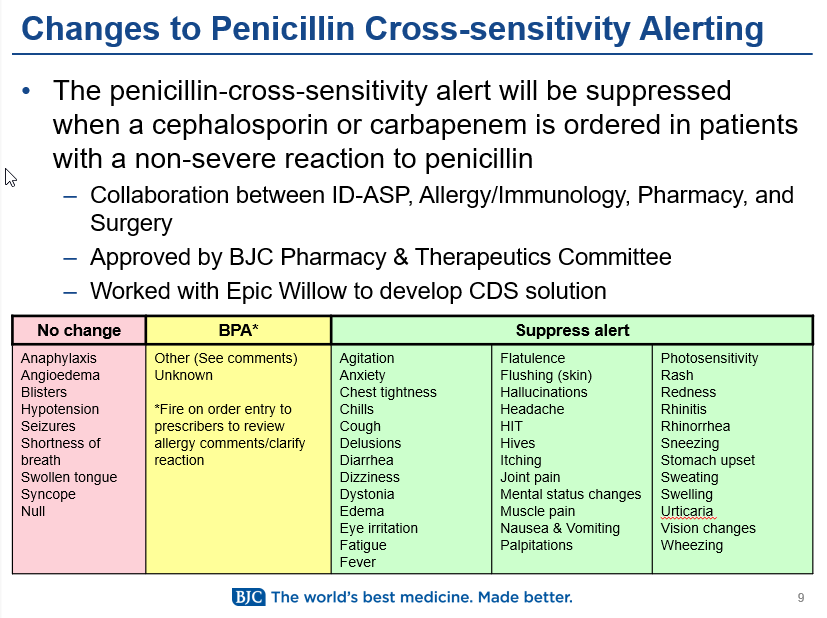

Supplement: Durkin et al. supplementary material [file S0899823X25103115sup001.docx]
